# Attaining ISO 15189 accreditation through SLMTA: A journey by Kenya’s National HIV Reference Laboratory

**DOI:** 10.4102/ajlm.v3i2.216

**Published:** 2014-11-03

**Authors:** Thomas Gachuki, Risper Sewe, Jane Mwangi, David Turgeon, Mary Garcia, Elizabeth T. Luman, Mamo Umuro

**Affiliations:** 1 Kenya Ministry of Health, National HIV Reference Laboratory, Kenya; 2 Division of Global HIV/AIDS, US Centers for Disease Control and Prevention, Nairobi, Kenya; 3 Division of Global HIV/AIDS, US Centers for Disease Control and Prevention, Atlanta, Georgia, United States; 4 Clinical Pathology Laboratories, Austin, Texas, United States

## Abstract

**Background:**

The National HIV Reference Laboratory (NHRL) serves as Kenya’s referral HIV laboratory, offering specialised testing and external quality assessment, as well as operating the national HIV serology proficiency scheme. In 2010, the Kenya Ministry of Health established a goal for NHRL to achieve international accreditation.

**Objectives:**

This study chronicles the journey that NHRL took in pursuit of accreditation, along with the challenges and lessons learned.

**Methods:**

NHRL participated in the Strengthening Laboratory Management Toward Accreditation (SLMTA) programme from 2010–2011. Improvement projects were undertaken to address gaps in the 12 quality system essentials through development of work plans, team formation, training and mentorship of personnel. Audits were conducted and the scores used to track progress along a five-star grading scale. Standard quality indicators (turn-around time, specimen rejection rates and service interruptions) were measured. Costs of improvement projects and accreditation were estimated based on expenditures.

**Results:**

NHRL scored 45% (zero stars) at baseline in March 2010 and 95% (five stars) after programme completion in October 2011; in 2013 it became the first public health laboratory in Kenya to attain ISO 15189 accreditation. From 2010–2013, turn-around times decreased by 50% – 95%, specimen rejections decreased by 93% and service interruptions dropped from 15 to zero days. Laboratory expenditures associated with achieving accreditation were approximately US $36 500.

**Conclusion:**

International accreditation is achievable through SLMTA, even for a laboratory with limited initial quality management systems. Key success factors were dedication to a shared goal, leadership commitment, team formation and effective mentorship. Countries wishing to achieve accreditation must ensure adequate funding and support.

## Introduction

The burden of HIV in Kenya is high, with 1.6 million people living with the infection as of December 2011, including 621 813 patients who had been placed on antiretroviral therapy (ART) by 2010. In order to support diagnostic testing and laboratory monitoring of HIV patients, there is a high demand for quality laboratory services, as 5.7 million HIV tests were performed in 2012 alone.^1^

Gershy-Damet et al. pointed out that high-quality laboratory testing is critical for patient care, disease prevention and surveillance.^2^ Laboratory test results play a crucial role in medical decision making; and accurate and reliable diagnostic testing and monitoring are critical to the successful management of HIV. In order to ensure the reliability and accuracy of testing, a quality system that addresses all aspects of testing is essential. However, establishing and maintaining high-quality testing standards presents major challenges in resource-poor settings.^3^ Key amongst these challenges is lack of adherence to international standards as a result of inadequate quality management systems (QMS)^4^ that focus on achieving quality testing services. In addition, because most HIV diagnostic testing is done by non-laboratory staff, reference laboratories play a critical role in monitoring field testing.^2,5^ To ensure quality results at every level, the World Health Organization (WHO) recommends that national reference laboratories seek accreditation to international standards.^6^

In 2003, the Kenya Ministry of Health established the National HIV Reference Laboratory (NHRL), a public health facility designed to monitor the quality of HIV testing by providing a serology proficiency scheme, conducting external quality assessment (EQA) testing and acting as the centre of excellence in the laboratory monitoring of HIV patients. Initially, the NHRL did not have QMS in place and was not benchmarking itself against international standards. The quality of analytical testing and services was not validated, limiting its ability and authority to act as a centre of excellence.

In 2010, the NHRL adopted the Ministry of Health’s goal to accredit all national and regional level public laboratories in Kenya to the International Organization for Standardization (ISO) 15189 standard, which is specifically designed to encourage medical laboratories to develop a highly disciplined approach to improving the quality of services.^7^ ISO 15189 assesses the competence of the QMS within the laboratory,^8^ provides a framework for increased analytical quality^9^ and verifies that laboratories are not deviating from quality and competency standards.^10^ The accreditation journey at the NHRL began in 2009 when laboratory management invited a consultant from A Global Healthcare Public Foundation (AGHPF) to review the current laboratory QMS and provide advice on needed improvements. The findings of this review stirred the management to seek assistance in the development and implementation of a more robust QMS.

In 2010, NHRL adopted the Strengthening Laboratory Management Toward Accreditation (SLMTA) programme and enrolled in Kenya’s first cohort along with 12 other laboratories, with the goal of attaining ISO 15189 accreditation.

This paper chronicles the journey that the NHRL took in the pursuit of international accreditation, along with the challenges and lessons learnt. We show how management commitment, team formation, culture change and mentorship were instrumental in the successful completion of this journey.

## Research method and design

### Study site

The NHRL is located in the capital city of Nairobi and consists of three main sections: serology, molecular, and ART monitoring. In addition, there are two cross-cutting sections: logistics, and monitoring and evaluation. Each section is managed by a team lead.

In its role as an HIV referral laboratory and centre of excellence, the NHRL is responsible for strengthening laboratory systems for HIV diagnosis, care, treatment and surveillance. It provides leadership and support to the national HIV laboratory programme by formulating policy and guidelines on HIV laboratory-related issues and coordinating activities and partners. The NHRL offers reference services in HIV testing and laboratory ART monitoring, including HIV viral load testing, early infant diagnosis, CD4 lymphocyte enumeration and the evaluation and monitoring of the quality of HIV testing reagents and equipment. It also provides and coordinates EQA services in HIV testing by running the national HIV Serology Quality Assurance Program for over 7000 laboratory and non-laboratory testing personnel. Additionally, the NHRL is responsible for EQA programmes in CD4 lymphocyte enumeration, haematology and chemistry. The NHRL also provides support and mentoring to HIV testing and ART monitoring personnel, as well as building in-country capacity to design, implement and evaluate HIV-related surveillance systems and surveys.

### SLMTA process and evaluation

The SLMTA programme uses the Stepwise Laboratory Quality Improvement Process Towards Accreditation (SLIPTA) checklist in order to identify strengths and weaknesses and to measure progress. The SLIPTA checklist provides an evaluation score based on laboratory quality in the 12 quality system essentials (QSEs). Laboratories are assigned a ‘star’ level based on their scores: zero stars (0% – 54%), one star (55% – 64%), two stars (65% – 74%), three stars (75% – 84%), four stars (85% – 94%), and five stars (≥ 95%). Laboratories that score five stars are encouraged to pursue ISO 15189 accreditation.^11^

A baseline audit was conducted in March 2010 by SLMTA in-country trainers using the SLIPTA checklist. This was followed by the first SLMTA workshop in April 2010, then the second workshop in September of the same year and the third workshop in January 2011. An exit audit was conducted by auditors from the Kenya Accreditation Service (KENAS) in October 2011.

In February 2012, a consultant from the South Africa National Accreditation Service (SANAS) performed a pre-accreditation assessment utilising the SANAS 15189 checklist in order to determine readiness for accreditation.

Several quality indicators were monitored weekly, monthly or annually so as to assess the impact of the SLMTA programme on laboratory service quality and patient care. Specimen turnaround times for viral load, enzyme-linked immunosorbent assay (ELISA) and CD4 tests were calculated using data from the laboratory information management system (LIS). Information on service interruptions because of equipment downtime and stockouts was obtained from the LIS monthly and averaged over a calendar year. Customer satisfaction was estimated from patient feedback forms that were availed either in laboratory reception areas or by mailing to customers. Specimen rejections were tallied from the LIS. Corrective actions and occurrence management were evaluated based on completed corrective action forms and quarterly reports. These were divided into three phases: pre-analytic, analytic and post-analytic. Routine results from EQA panel tests for all analytes were collated and performance evaluated using Microsoft^®^ Excel 2007, by aggregating the score achieved in every EQA challenge and obtaining a percentage score. A score of 100% was desirable, whilst any score below this would call for corrective action.

Costs of programme implementation were estimated in US dollars based on expenditures made by the laboratory on quality improvement. These costs include fees paid to the accrediting body, KENAS, and the cost of various improvements such as access control, safety equipment, equipment service contracts, ISO training, EQA enrolment, storage area renovation and electronic temperature-monitoring system. For this analysis, in-kind contributions such as mentorship provided by the US Centers for Disease Control and Prevention (CDC) and costs borne by the Ministry of Health, such as SLMTA training, were not included. The opportunity cost of staff time to participate in training, complete the improvement projects and prepare for accreditation was also not included.

### Team formation

To implement the QMS, a strategic, tiered, accreditation team structure with a clear reporting mechanism was formed. The structure included a Management Team, a Quality Assurance (QA) Team and Section Teams.

The Management Team was composed of the laboratory manager, deputy laboratory manager/QA manager, Section Team leads, the safety officer and the logistician. This core group guided the accreditation process and held regular review meetings in order to track progress and monitor the quality indicators adopted by the laboratory. They also reviewed gaps identified in both internal and external audits and formulated plans for continuous quality improvement.

The QA Team, reporting to the Management Team, was chaired by the QA manager/deputy laboratory manager and two QA officers (one also serving as the safety officer). This team was responsible for monitoring the accreditation process, offering leadership and coordinating the implementation of various improvement projects. Each member of this team was assigned to a section and mentored by the QA manager.

Section Team leads were given authority to make decisions and were ultimately responsible for improvement projects within their section. The Section Teams held weekly meetings to discuss problems and possible solutions and to track the progress of improvement projects within their section. Section Team leads reported to the QA team on all critical issues pertaining to QMS implementation.

Annual staff retreats were held at the beginning of each year, during which work plans were developed with clear timelines and action points that incorporated all 12 QSEs and were based on ISO 15189 requirements. Team building also took place during the annual staff retreats. These plans were posted on bulletin boards where they were visible to all staff. Regular monthly staff meetings were held in order to review work plans and monitor progress of the quality improvement initiative. After every internal and external audit, work plans were modified so as to reflect progress made and to redirect efforts where needed.

Individual staff members set annual accreditation goals and targets against which they were appraised for their annual staff performance contracts. An employee recognition scheme was put in place and incentives were provided. Laboratory management led the way by prioritising accreditation and making sure that all personnel were keenly aware of the accreditation goal; accreditation was the main agenda item in all meetings and took priority in budget considerations, ensuring that resources required for the process were secured.

### Improvement projects

Improvement projects were undertaken for all 12 QSEs in order to address the gaps identified in the audits. Each member of the NHRL staff was responsible for at least one project with clear timelines. The findings of routine audits were used to make continual improvements within the QMS. The laboratory undertook more improvement projects (Table 1) than required by Kenya’s SLMTA team, including changes to the design of the laboratory and development of workflow diagrams. The plan–do–check–act cycle was adopted in implementation of the quality improvement projects.^12^ Most importantly, method validation was performed in order to assess the methods and equipment utilised in the laboratory.^13^

**TABLE 1:** Gaps identified and corresponding improvement projects conducted for SLMTA implementation at Kenya’s National HIV Reference Laboratory, 2010–2013.

Quality System Essential	Gap Identified	Planned Improvement Project	Indicator	Outcome
**Organisation**	No legal identity	Register with the Kenya Medical Laboratory and Technologists Board	Kenya Medical Laboratory and Technologists Board registration certificate on file	Registered with the Kenya Medical Laboratory and Technologists Board
	No organogram	Develop organogram	Organogram in place	Organogram developed
	No minutes for staff meetings	Develop minutes template	Staff meeting minutes template in place	Minutes template developed and implemented
	No management review of the effectiveness of the quality management system	Develop procedure and hold management review meetings	Procedure and minutes of management review meetings in place	Management review of meetings held
	No deputies for key personnel	Appoint deputies for key personnel	Deputy appointment letters in place	Deputies for key personnel appointed
**Documents and records**	No policy on document control or sample retention	Develop policies and procedures for document control and sample retention	Document control and sample retention policies in place	Document control and sample retention policy and procedure developed and implemented
	No quality manual or standard operating procedures	Develop quality policy manual and standard operating procedures	Quality policy manual in place	Quality policy and standard operating procedures developed
	Test method procedures required additional information for compliance	Adopt standard operating procedures template based on ISO recommendations	Standard operating procedure template in place	Standard operating procedures template based on ISO recommendations developed and adopted
**Facilities and safety**	No safety officer	Appointment of safety officer	Safety officer appointment letter and job description on file	Safety officer appointed
	No safety manual	Development of safety manual	Safety manual adopted	Safety manual developed
	Not secured from unauthorised access	Procure and install biometric access	Biometric access installed	Biometric access control put in place
	Lack of appropriate safety signage	Post appropriate safety signage	Safety signage posted	Safety signage and floor plan posted
	No contract with external contractor who disposed of infectious waste	Develop contract with external waste disposal contractor	Contract in place	Contract with waste disposal firm created
	Standard safety equipment not available/not routinely serviced	Procure and maintain standard safety equipment	Safety equipment in place	Safety equipment procured and maintenance schedule developed
	Staff not immunised	Immunise staff	Staff immunisation records on file	All staff immunised
	No post-exposure prophylaxis guidelines	Develop post-exposure prophylaxis guidelines	Post-exposure guidelines in place	Post-exposure guidelines developed
	Couriers not trained on safety	Train couriers on safety points	Training records for couriers on file	Couriers trained on safety
	Electrical safety not observed	Develop procedure on electrical safety and add electrical access points	Procedures on electrical safety adopted and additional electrical points installed	Procedures on electrical safety developed and additional electrical access points installed
	No Material Safety Data Sheets	Download and develop Material Safety Data Sheets	Material Safety Data Sheets in place	Material Safety Data Sheets developed
**Personnel**	No personnel files	Develop personnel files	Personnel files in place	Personnel files created
	No orientation records	Develop procedures for staff orientation	Orientation records on file	Orientation for staff completed
	No job descriptions	Develop and issue job descriptions	Job descriptions on file	Personnel given job descriptions and appointment letters
	No competency assessment records	Develop and implement competency assessment procedures	Competency assessment records on file	Competency assessments conducted for all personnel
	No work or bench schedules	Develop work and bench schedules	Work and bench schedules in place	Standardised duty roster stating work schedules developed
	No employee satisfaction surveys conducted	Develop and implement procdures for employee satisfaction surveys	Employee satisfaction survey records on file	Employee satisfaction surveys completed
**Purchasing and inventory**	No inventory system in place	Develop inventory system in the laboratory information system	Inventory system module in place in the laboratory information system	Inventory system developed in the laboratory information system
	No list of approved suppliers	Develop procedures for evaluation of suppliers	Procedure for evaluation of suppliers and list of approved suppliers in place	Suppliers evaluated and approved suppliers list created
	Lack of proper storage area	Renovate an extra room and convert into a storage area	Storage area available	Extra room space renovated and converted into a storage area
	No protocol for disposal of expired products	Develop and implement protocols and procedures for disposal of expired products	Protocols and procedures for disposal of expired products in place	Protocols and procedures for disposal of expired products developed and implemented
**Process control and equipment**	Work processes not defined and task schedules not documented for specific departments or their staff	Develop work schedules	Work schedules in place	Work schedules developed
	Environmental checks, e.g. room temperature, not monitored	Develop procedures for environmental checks and procure room temperature thermometers	Room temperature thermometers in place Procedures for environmental checks in place	Room temperature thermometers procured
	Field staff not trained on sample management	Develop procedures for sample management and train field personnel	Procedures for sample management in place	Field personnel trained on sample management
			Field personnel training records on file	
	No recording of patient’s date of birth, gender or initials of collector during sample collection	Adjust patient request and report forms to meet ISO requirements	Patient request and report forms that meet ISO requirements in place	Changes implemented on the LIS system in line with ISO requirements for patient request and report forms
	Equipment had no unique identifiers/inventory data	Develop equipment inventory and procedures/protocols for method validation	Equipment inventory and method validation records on file for all methods and equipment	Equipment inventory developed, method validation performed for all methods and equipment
**Process control and equipment**	No schedule of service for most equipment	Develop service schedules for all equipment	Service schedules in place	Service schedules developed for all equipment
	Freezer/refrigerators not monitored consistently	Procure electronic system to monitor all refrigerators, freezers and environmental temperatures	Electronic monitoring system in place	Electronic system to monitor all the refrigerators, freezers and environmental temperatures procured
	Back-up equipment insufficient, i.e. power generator continually fluctuated on and off	Procure new generator	Functional back-up generator in place	New generator procured
	No procedures for handling specimens during equipment failure	Develop a back-up policy and procedures	Back-up policy and procedures in place	Back-up policy and procedures developed
	No calibration of timers, thermometers, pipettes and readers	Calibrate all timers and thermometers	Calibration records for timers and thermometers on file	Develop calibration records for all timers and thermometers
	No validation of test methods	Develop protocols and validate all test methods	Test method validation records on file	All test methods were validated
	No quantitative analysis using Westgard rules	Develop procedures for quantitative analysis using Westgard rules for every method and plot LJ charts	Procedures for LJ chart plotting and monitoring in place	Procedures for quantitative analysis using Westgard rules for every analysis developed and LJ charts plotted and monitored
	No lot-to-lot monitoring of new test kits	Develop procedures for lot-to-lot validation	Procedures and records for lot-to-lot validation in place	Procedures for lot-to-lot validation developed and implemented
**Information management**	No automatic back-up system, no controlled environment for server	Develop procedures for data back-ups	Back-ups conducted daily	Procedures for data back-ups developed and implemented; daily back-ups conducted
	Some data were backed up on hard discs but stored in wooden cabinets; no fire-proof cabinets available	Procure fire-proof cabinets	Fire-proof cabinets in place	Fire-proof cabinets procured
	No monitoring of quality indicators	Make changes on LIS to monitor quality indicators such as turnaround times, specimen rejection, staff productivity, service interruptions	Records of monitored quality indicators on file	Improvements made on LIS to monitor quality indicators such as turnaround times, specimen rejection, staff productivity, service interruptions
	Results released without quality assurance reviews	Introduce three levels of review of results on LIS	Results of review records on file	Three levels of review of results introduced on LIS; LIS used to email results directly to clients
**Occurrence management**	No documentation of corrective action	Develop corrective action policy, procedure and log	Corrective action log in place	Corrective action policy and procedures developed Corrective action log developed
	No evidence that laboratory performance was monitored and non-conformities identified and closed; no documentation of corrective actions from external audits	Perform quarterly analysis of occurrence management	Ocurrence reports on file	Quarterly analysis of occurrence management conducted and presented to management for review
	No communications book in any department	Develop procedures for communication and introduce communications books	Communications books in place	Communications books introduced in all departments; regular reviews by management conducted
**Assessments**	No schedule for internal audits; internal audits not carried out	Develop procedures and schedule for internal audits	Schedule and internal audits reports on file	Schedule for internal audits developed, and performed regularly
	No internal auditors	Train internal auditors	Internal auditors in place	Internal auditors trained
	No participation in external quality assessment for all methods	Develop policy and procedures on external quality assessment and enrol all sections	Policy and procedures in place and molecular section enrolled in an external quality assessment program	Policy and procedures developed; Molecular section enrolled in an external quality assessment programme
	Internal quality control not monitored or reviewed	Develop procedures for internal quality control	Internal quality control chart in place	Chart to monitor internal quality control developed and regularly reviewed by supervisor
**Customer service**	No system or schedule for evaluating customer satisfaction	Develop policy and procedures and perform customer satisfaction surveys	Customer survey records on file	Customer satisfaction policy and procedures developed and surveys performed
	No handbook outlining the lab’s activities for its clients, i.e. hours of operation, available tests, turnaround time for tests	Develop laboratory handbook	Laboratory handbook in place	Laboratory handbook developed
**Process improvement**	No improvement projects were undertaken by the laboratory	Develop policy and procedures for continual improvement process	Policy and procedure projects in place	Policy and procedures for continual improvement projects developed
	No QA reports	Develop policy and procedures for QA reports	QA report in place	Develop QA report on all improvement projects for Director
	No evidence of supervisor review	Monthly review of improvement projects	Monthly review of improvement project reports in place	Monthly review of improvement project documentation by QA team

SLMTA, Strengthening Laboratory Management Toward Accreditation; ISO, International Organization for Standardization; LIS, Laboratory Information Management System; QA, Quality Assurance; LJ, Levey-Jennings.

All staff members were actively involved in the quality improvement projects. Work plans were developed at the beginning of each year and after every audit. The work plans involved establishing a strategic goal and objective, with responsibility and project timeline assigned. Work plans were reviewed regularly in staff meetings and were located centrally in the laboratory for easy reference. The work plans served as valuable tools for setting realistic targets, measuring progress and enforcing individual responsibility, leading to a focused implementation of improvement projects. Flow diagrams were developed to assist in identifying weak areas and making necessary improvements.

### Training

All laboratory personnel were trained on the ISO 15189 standard by Management Sciences for Health and on Good Laboratory Practice by the Kenya Aids Vaccine Initiative. Staff also underwent 14 days of mentorship training in three of Kenya’s internationally-accredited research laboratories: the Kenya Medical Research Institute (KEMRI)/CDC HIV Research Laboratory in Kisumu, the US Army Walter Reed Laboratory in Kericho and the Academic Model for Treatment Laboratory in Eldoret. The QA manager also attended internal audit training conducted by SANAS.

### Mentorship

Two mentors from CDC’s International Laboratory Branch, Division of Global HIV/AIDS in Atlanta spent a total of eight weeks in the NHRL during the SLMTA process. An initial three-week visit was made in January 2011 following the second SLMTA workshop. To make effective use of the mentors’ time on site, a brief report was prepared by the NHRL in advance of the first visit and shared with the mentors, including information on test methods and equipment used in the NHRL. At the beginning of the visit an internal audit was performed and a work plan developed based on the findings, in collaboration with the QA team and individual members of the various laboratory sections. At the end of the visit another audit was performed and the entire team participated in development of another work plan for outstanding issues.

Long-distance support then followed via email for a six-month period. An additional two-week visit was made by one of the mentors, who is also a member, inspector and team lead for the College of American Pathologists. A final three-week visit was made by both mentors in January 2012, three months after the exit audit, in order to prepare the laboratory for the ISO accreditation pre-assessment.

## Results

### Audit scores and accreditation

At the baseline audit in March 2010 before SLMTA implementation, NHRL scored 45%, corresponding to zero stars. At the October 2011 exit audit, the laboratory more than doubled their score to 95%, earning five stars. In March 2013, three years after initiation of SLMTA, the NHRL achieved accreditation to ISO 15189.

### Improvement projects

Gaps were identified in all 12 QSEs after the baseline audit. Improvement projects were undertaken to address these problems (Table 1). Some projects were one-time activities, such as development of policies and procurement; for example a policy on environmental control was developed and room thermometers were procured. Other projects implemented more comprehensive on-going changes to laboratory procedures, such as quarterly analysis of occurrence management and keeping minutes at staff meetings. All the improvement projects that were undertaken were completed by the time the laboratory attained accreditation.

### Quality indicators and costs

Average turn-around time for viral load testing decreased from 20 days in 2010 to six days in 2013 (70%). Similarly, ELISA turn-around time decreased from 191 days to 10 days (95%). CD4 turn-around time decreased from 24 hours to 12 hours (50%). The number of rejected specimens decreased from 133 in 2010 to nine in 2013 (93%) and the number of service interruption days decreased from 15 to zero (100%) (Table 2).

**TABLE 2:** Trends in quality indicators, Kenya’s National HIV Reference Laboratory, 2010–2013.

Quality indicator	2010	2011	2012	2013	Improvement (2010 to 2013) %
**Average turn-around time**					
Viral load (days)	20	14	10	6	70
ELISA (days)	191	62	46	10	95
CD4 (hours)	24	24	12	12	50
Specimen rejections (no. of specimens)	133	15	22	9	93
Service interruptions* (days)	15	5	0	0	100
Customer complaints from patient satisfaction surveys (no.)	12	3	3	5	58
**Corrective actions and occurrence management (no.)**					
Pre-analytical	31	20	13	12	61
Analytical	29	13	10	9	69
Post-analytical	14	8	3	5	64
EQA performance** (%)	60	80	100	100	40

CD4, cluster of differentiation 4; ELISA, enzyme linked immunosorbent assays; EQA, external quality assessment;

* Because of equipment down-time and stock-outs;

** Percentage of overall score.

The cost to the laboratory to conduct SLMTA improvement projects and to continue through to ISO 15189 accreditation was US$36 500 (Table 3).

**TABLE 3:** Expenditures by Kenya’s National HIV Reference Laboratory to achieve ISO 15189 accreditation

Item	Sub Level	Cost ($)
SLMTA workshops	-	Donation In kind^1^
Mentorship, Atlanta	-	Donation In kind^2^
Consultants	-	Donation In kind^1^
Accreditation fees, KENAS	-	7000
Improvement projects	Access control	1000
	Safety equipment (eye wash stations, emergency showers, spill kits, fire extinguishers, fire alarm, first aid kits)	1000
	Fire proof cabinets	500
	Equipment service contracts	3000
	Back-up generator	10 000
	GCLP training	Donation in kind^1^
	ISO training	Donation in kind^3^
	Staff mentorship in accredited laboratories	2000
	Staff immunisation	Donation in kind^4^
	EQA providers	1000
	Storage area renovation	6000
	Electronic temperature-monitoring system	5000
**Total**	**36**	**500**

SLMTA, Strengthening Laboratory Management Toward Accreditation; KENAS, Kenya Accreditation Service; ISO, International Organization for Standardization; GCLP, Good Clinical Laboratory Practice; EQA, external quality assessment.

$, US Dollars.

1 Division of Global HIV/AIDS, US Centers for Disease Control and Prevention (CDC), Nairobi, Kenya through cooperative agreement with the government of Kenya.

2 Division of Global HIV/AIDS, CDC, Atlanta, Georgia, United States.

3 Management Sciences for Health (MSH).

4 Division of Vaccine, Kenya Ministry of Health.

## Discussion

The NHRL was successful in achieving accreditation to ISO 15189 in March 2013, three years after beginning the quality improvement process. High-quality laboratory testing is critical for patient care, disease prevention and disease surveillance.^5^ Although the majority of laboratory testing is done by public laboratories, no laboratory in the public sector had been accredited previously in Kenya, as all eight accredited laboratories were private or research laboratories. In fact, in all of sub-Saharan Africa except South Africa, only two public laboratories had been accredited previously to international standards: one in Namibia and one in Botswana.^14^

The success of NHRL was a result of several factors. Firstly, the team was built with a shared vision, all striving to meet ISO 15189 requirements. Collective involvement has been shown elsewhere to be important in implementing change.^15,16^ The SLMTA trainees shared their projects with all staff, who then took up responsibility; this helped to prevent the mentality that quality improvement was ‘someone else’s job’ and ensured shared ownership of the process. In the weekly section meetings, brainstorming led to development of local solutions and sharing of best practices, ensuring there was no slackening of momentum. These meetings also enhanced the cohesiveness of the entire NHRL staff team.

Secondly, the old adage is true: what gets measured, gets done. SLIPTA scores and star levels provided a framework for identifying strengths and weaknesses and quantifying progress. The baseline audit offered an objective analysis of processes in the laboratory, revealed critical gaps in the system and guided the team in initiating a gradual process of preparedness for accreditation. The exit audit documented how far the laboratory had come, giving leadership and staff the motivation to continue improving and the confidence to seek international accreditation.

Thirdly, the SLMTA programme provided NHRL staff the training needed to make QMS improvements quickly and to prepare for accreditation. The laboratory used SLMTA improvement projects as a springboard to implement additional projects with a wider scope in order to cover all aspects of the QMS. Changes to the design of the laboratory and workflow diagrams allowed efficient and logical flow of work processes. Improvement in testing turn-around time was achieved by preventing service disruptions, ensuring uninterrupted reagent supply, establishing equipment service contracts and creating a back-up programme.

Fourthly, mentorship was key in helping the laboratory customise solutions. Effective mentorship has been shown to be a success factor in the implementation of SLMTA in various settings.^16,17^ The two CDC mentors not only spent periods of time in the facility but also offered guidance and assistance remotely. The intense preparation conducted by laboratory staff before the visits enhanced focus and sustainability when the mentors left. The mentors did not perform tasks, but instead guided laboratory staff to do them, fostering ownership and building capacity. Contact was maintained with mentors after they left, ensuring continuity. The mentees identified high-priority areas in which they required assistance, saving time onsite. A positive staff attitude facilitated the productive relationships with mentors; no time was wasted in finding common ground because all shared the same goal of NHRL accreditation.

Finally, continued focus on accreditation after SLMTA allowed the laboratory to reach even higher levels. The pre-accreditation assessment conducted by the SANAS assessor offered an objective in-depth analysis using a different checklist and gave laboratory staff an idea of what to expect in the accreditation visit. Findings from this assessment were used to address remaining gaps prior to the official inspection.

NHRL faced many critical challenges in implementing QMS, as summarised in Table 4. One serious problem that remains unsolved is staff attrition. Because the government handles staff deployment, trained staff members are often transferred to other laboratories. NHRL is working with the Ministry of Health to prioritise continuity of staff and training for new staff members in order to sustain quality levels.

**TABLE 4:** Challenges and solutions for quality improvement, Kenya’s National HIV Reference Laboratory, 2010–2013.

Challenge identified	Solution
Staff thought that the accreditation mandate belonged to the QA manager alone	Change in staff culture and attitude resulted from a three pronged approach: mentorship in accredited laboratories, training on ISO 15189, and training on Good Clinical Laboratory Practice. As a result, staff were now knowledgeable on what was required, best practises, and the benefit of accreditation. All staff were involved in selecting and managing improvement projects. This made it easier for everyone to embrace the quality management system.
Lack of knowledge on ISO 15189 standard requirements	All laboratory staff received training on ISO 15189 and Good Clinical Laboratory Practice. Everyone was also given a personal copy of the ISO standard, and were challenged to refer to it often to identify issues that they could help resolve.
Staff concerns about filling out corrective action forms and occurrence management reports because they thought of them as punitive	The training on ISO helped staff understand the importance of occurrence management. This was reinforced by involving them in revising the existing corrective action form followed by training by the Quality Assurance Team. Staff were reassured that the forms and reports would be used for improvement only, and would not be used against them.
Procurement process was slow, delaying implementation of projects	Staff learned to plan ahead and place orders with long lead times.
Development of method validation protocols for each test method is complicated	Method validation training was provided to all staff, including training on accuracy, precision, and reportable ranges.
Various experts and mentors had contradicting styles and opinions	Early in the process, the laboratory selected two mentors that they used exclusively for the duration of the process. Proper engagement structures were set in place for stakeholders and support partners.
Major safety deficiencies and shortage of space	Due to shortage of space, the laboratory was borrowing storage space over which it did not have control. It was therefore difficult to set up emergency exits and dedicated areas for freezers and fridges. Permanent space was eventually acquired in nearby facilities.
Lack of accredited public laboratories to use as back-up (private accredited laboratories would require payment)	A checklist for evaluation of nearby public laboratories was developed to help identify and prepare other laboratories to perform back-up services.

QA, Quality Assurance; ISO, International Organization for Standardization.

The NHRL spent approximately US$36 500 in pursuit of ISO 15189 accreditation, in addition to that spent by the Ministry of Health on SLMTA training and by partners for mentorship and additional training. One of the largest expenses was the placement of equipment on service contracts. To reduce costs, the laboratory adopted the equipment placement model, whereby an equipment manufacturer places equipment in a laboratory at no cost, recovering their expenses by selling reagents to the laboratory. Other substantial expenses included the renovation of a storage room to overcome space shortages and installation of a temperature-monitoring system in order to improve the archiving of specimens. The largest single expense was the purchase of a back-up generator; this purchase also benefited other users within the National Public Health Laboratories complex. Many key components of the programme were paid for by various partners and were thus not included in the cost estimate. For example, ISO training was sponsored by Management Sciences for Health and included staff from other laboratories. Personnel were immunised by the Division of Vaccination in the Ministry of Health. Finally, the AGHPF consultant and CDC mentors, critical for readying the laboratory for accreditation, were sponsored by their respective organisations.

Cost considerations must be weighed against the benefits of quality improvement. Some improvements will result in large cost savings over time. Human resource management has been made easier as staff competencies are assessed annually; personnel are now more efficiently assigned to specific responsibilities based on their core competencies. The process of HIV results confirmation, which used to take more than one month, now takes less than 10 days, ensuring rapid resolution nationwide for clients with discrepant HIV results. HIV viral load results are now received in less than 10 days; this information is critical with regard to alerting clinicians to the need to change treatment regimens for patients with treatment failure, thus reducing their likelihood of developing drug resistance. Services are no longer interrupted because of reagent shortages or equipment downtime and adherence to sample handling guidelines has greatly reduced rejected samples, decreasing both costs and wastage.^18^ Accreditation also provides immeasurable benefits in enabling the NHRL to fulfil its mission as the country’s reference laboratory for HIV testing. It has accorded the NHRL international recognition and elevated customer confidence with respect to the reliability of services as they fulfil their mandate. Pursuit of accreditation has led to significant improvement in the quality of both analytical test results and customer service. Because of the central role the laboratory plays in Kenya, these benefits have a direct impact on the quality of HIV testing and monitoring throughout the country.

### Conclusion

The experience of Kenya’s NHRL shows that it is feasible to attain international accreditation through the implementation of the SLMTA programme, even in settings with poor resources and laboratories without initial systems.
